# The mediating role of social support in behavioral changes and weight loss outcomes among overweight Appalachian adults

**DOI:** 10.1007/s10865-025-00555-0

**Published:** 2025-02-09

**Authors:** Xiaochen Zhang, Abigail Shoben, Ashley S. Felix, Brian C. Focht, Ryan D. Baltic, Electra D. Paskett

**Affiliations:** 1 Division of Cancer Prevention and Control, Department of Internal Medicine, College of Medicine, The Ohio State University, 3650 Olentangy River Road, Columbus, OH USA; 2 Division of Biostatistics, College of Public Health, The Ohio State University, Columbus, USA; 3 Division of Epidemiology, College of Public Health, The Ohio State University, Columbus, USA; 4 Kinesiology, Department of Human Sciences, The Ohio State University, Columbus, USA

**Keywords:** Weight loss, Obesity, Social support, Appalachian, Health disparities

## Abstract

Social support plays a key role in behavioral changes, especially in Appalachian populations. We examined the mediating effect of social support in behavioral changes and corresponding weight loss outcomes among Appalachian adults. Data were from a group-randomized trial that compared a 12-month faith-based weight loss intervention to an active control group among overweight Appalachian adults in churches. Participants from the weight loss intervention who completed the 12-month assessment were the focus of this analysis. Baseline and 12-month data on weight, social support for eating habits (SSEH) and physical activity (SSPA) from family, friends, and church family, physical activity, and dietary intake were collected. Logistic and linear regression models evaluated mediating effects of SSEH and SSPA on the association between intervention attendance and behavioral changes and corresponding weight loss outcomes. Most participants (*n* = 243) were female (76.2%), white (97.5%), and married or living with a partner (81.2%). After the 12-month intervention, participants lost weight (1.1 ± 0.3 kg), increased fruit and vegetable intake (0.4 ± 0.1servings/day), reduced caloric intake (322.9 ± 42.2 kcal/day), improved SSEH from family, and increased SSPA from the church family (all *P* < 0.05). Increased SSEH from family mediated 62% of the association between intervention attendance and fruit and vegetable servings per day. Each 100 kcal decrease in caloric intake was associated with decreased weight and BMI at 12-months (0.2 ± 0.1 kg, *P* = 0.003; 0.1 ± 0.02 kg/m^2^, *P* = 0.002). Our study demonstrated the mediation effect of social support for healthy eating on the association between intervention attendance and fruit and vegetable intake, which underscored the critical role of social support and calorie intake among Appalachian populations in losing weight. The study was pre-registered at clinicaltrials.gov (#NCT02121691).

## Introduction

Appalachia is a federally designated region across 13 states and ranges from southern New York to northern Mississippi (‘The Appalachian Region. ‘). More than 26 million people live in this region (Pollard, Srygley, and Jacobsen 2023). Residents in Appalachian regions experience significant obesity-related disparities. Compared to the national level, obesity-related disease-specific mortality, including cardiovascular disease, stroke, diabetes, and cancer are higher in the Appalachian regions (Marshall 2017). Further, Appalachian residents are more likely to be physically inactive compared to the national level (Rice et al., 2018; Marshall 2017).

Socioeconomic and environmental disadvantages, including low-income, limited resources and availability related to health and health care, transportation barriers, geographic isolation, and lack of awareness and skills could explain obesity-related disparities in Appalachian populations (Commission, 2012; Rodriguez et al., 2018; Wewers et al., 2006; Michimi and Wimberly 2010; Commission, 2017). However, Appalachian residents have unique characteristics, such as unhealthy social norms, familial influences, cultural practices, and lack of social support, which also leads to unfavorable health behaviors and obesity-related outcomes (Han, Moser, and Klein 2007; Niederdeppe and Levy 2007; Goins, Spencer, and Williams 2011; Schoenberg et al., 2013; Kobayashi and Smith 2016).

Although evidence-based weight loss programs have shown favorable health outcomes in clinical settings, these programs solely focused on health-promoting resources available in urban areas (Group 2013; Irwin, 2014; Brown et al., 2018; Kramer et al., 2009; Group, 2002; Sjöström et al., 2000; Stevens et al. 2001; Blumenthal et al., 2000; Ligibel et al., 2017; Morey et al., 2009; Goodwin et al., 2014; Matthews et al., 2017). Utilizing the same intervention strategies from these programs may not be feasible or effective for Appalachian populations. Previous studies showed residents in Appalachian areas had limited access to healthy food and exercise facilities and lack of social support from family and friends (Johnson et al., 2014; O’Brien et al., 2014; Hardin-Fanning, 2013). Social support is linked to improved self-efficacy and behavioral changes (e.g., increasing physical activity and healthy eating) (Anderson et al., 2007, 2010). Positive social support improves adherence to health-behavior change programs, psychological well-being, and weight management (Duncan & McAuley, 1993; Anderson-Bill et al., 2011). Group-based behavioral weight loss programs highlight the importance of support from peers and family, fostering a sense of community which is essential for maintaining behavioral changes(Jøranli et al., 2023). While both perceived and received support are beneficial, received support has a more significant impact on weight loss outcomes (Yan, 2020). However, negative social support, such as sabotage or passive behaviors, can hinder weight management efforts (Ogden & Quirke-McFarlane, 2023). Thus, understanding the role of social support on behavioral changes in a lifestyle intervention in Appalachian populations can inform future studies to design tailored intervention strategies to improve health behaviors and outcomes in this region.

Previous research has shown a positive relationship between behavioral change and weight loss outcomes, indicating that higher doses of behavioral interventions are linked with greater weight loss, thus, highlighting the significance of behavioral techniques in achieving sustainable weight loss (Burrell, 2023; Fitzpatrick et al. 2014; Perri et al., 2014). Nevertheless, the dose-response relationship between behavioral change and weight loss is unknown among Appalachian adults. It is essential to consider the ways behavioral change relates to weight loss outcomes across different populations and the best approach to determine the optimal behavioral changes (e.g., increasing physical activity, healthy eating) to achieve substantial weight loss. Understanding these nuances can help the design and tailoring of interventions that are culturally and contextually relevant to Appalachian populations, ultimately leading to a more effective weight loss program for this population. The objectives of the current study were all exploratory and aimed to (1) determine the mediation effect of changes in social support on the association between intervention adherence and behavioral changes; and (2) quantify the dose-response relationship between behavioral changes and weight loss outcomes among Appalachian adults with overweight/obesity.

## Methods

### Study design and population

This was a secondary analysis using data from a faith-based group-randomized controlled study, The Walk by Faith Trial, conducted in five Appalachian states (Baltic et al., 2015). Briefly, the Walk by Faith Trial recruited 663 participants from churches located in Appalachian counties from Ohio, Kentucky, Pennsylvania, Virginia, and West Virginia. Each state contained two geographic regions (county or group of counties), one randomized to the Walk by Faith (WbF) intervention and the other to the Ribbons of Faith (RoF) comparison group. Across the five states, a total of 13 churches were assigned to the WbF group, and 15 churches were assigned to the RoF group (Baltic et al., 2015; Paskett et al., 2018). Church members with a body mass index (BMI) > 25 kg/m^2^ from the participating churches in the five states were recruited to participate. The Ohio State University Institutional Review Board approved the study (#2011C0092). Informed consent was obtained from all participants prior to any study activity.

The WbF group focused on environmental and individual-level behavioral changes to reduce overweight/obesity (Baltic et al., 2015). Changes at the church level used social support and social networks to encourage behavioral change. Specifically, church navigators acted as role models by consistently wearing pedometers and provided encouragement and support by organizing group events and helping participants upload pedometer steps before and after services. Success stories were shared with participants at group events and from the pulpit, inspiring and motivating others to make behavioral changes. This sharing of experiences created a sense of connection among participants, ensuring they were not alone in the journey of behavioral change. Church navigators marked walking paths near the church with signage indicating distances traveled (0.25, 0.5, and 1 mile). Participants were encouraged to use a sign-up sheet placed within the church to share availability and interest in participating in walking groups, allowing church navigators to help match individuals with walking partners who had similar schedules. This facilitated increased walking time and strengthened social support within the church community. Other strategies included group activities such as healthy cooking challenges, physical activity challenges, healthy potlucks, and recipe swaps, which fostered a supportive environment where participants could engage in healthy behaviors together. Participants had access to a website designed for the intervention and monitored by research staff, where members could interact with one another, find an exercise group, and share healthy recipes. At the individual level, by offering interactive educational sessions on the computers, participants were asked to increase physical activity (primarily by walking), increase fruit and vegetable and water intake, reduce sugary drink consumption, and reduce dietary fat. Monthly sessions (one hour in length) at each church provided educational and motivational materials to participants.

The RoF comparison group focused on environmental and individual-level behavior changes to increase cancer screening knowledge and promote cancer screening, as recommended. Participants were also provided an informational session, a health fair, cancer education inserts in church bulletins, and monthly education sessions (one hour in length, cancer-related topics).

**Fig. 1:**
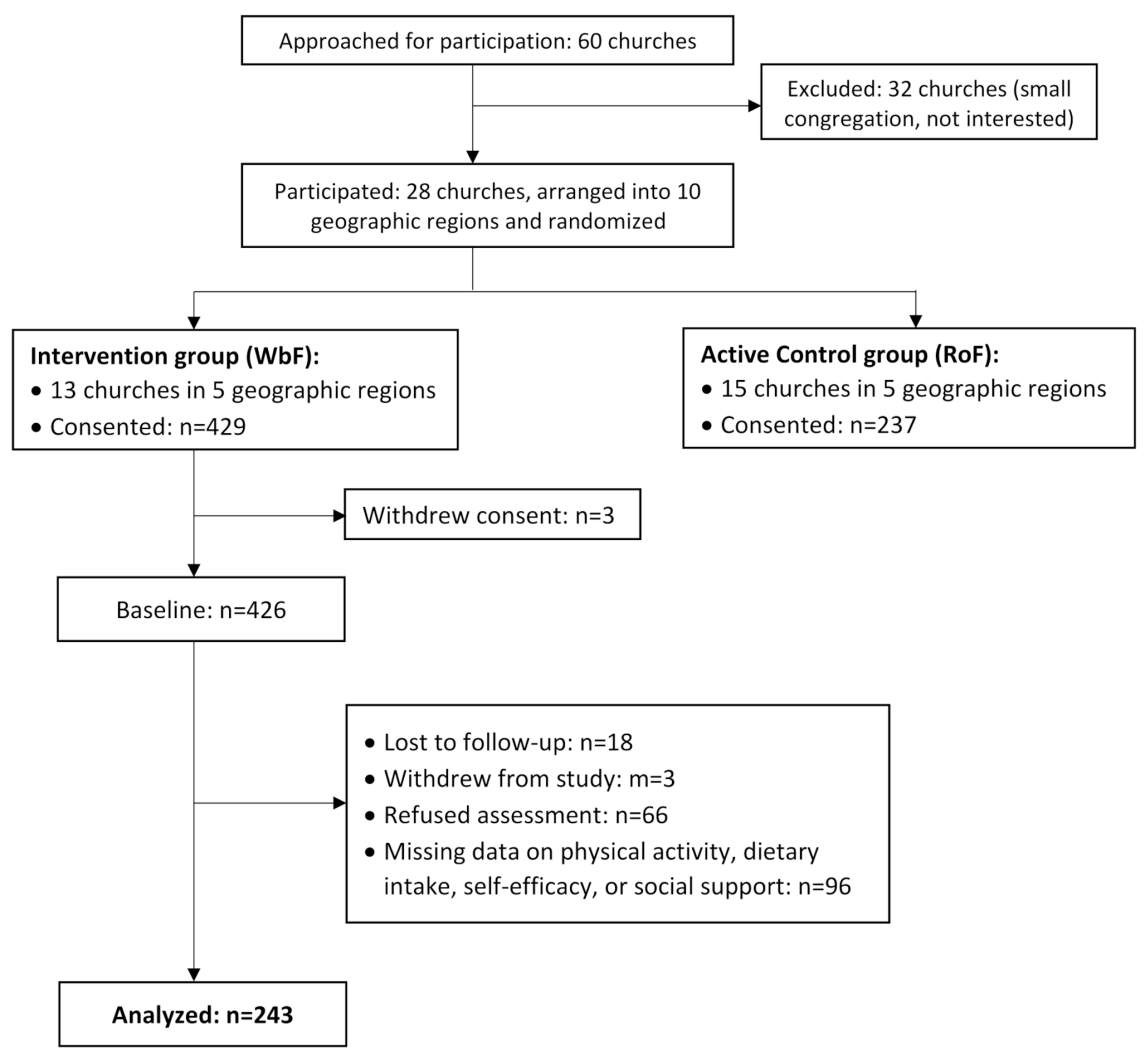
Walk by Faith study CONSORT diagram

Of the 344 church members screened for the RoF group, 237 (68.9%) consented to participate. Of the 525 church members screened for the WbF group, 426 (81.1%) consented and participated in the trial. At 12-months, 185 (78.1%) Rof and 289 (68.8%) WbF participants completed biometric assessments. Compared to RoF, participants in WbF lost 1.4% body weight (*p* = 0.13) and increased 35.4% MET-min/week physical activity (*p* = 0.056) from baseline, and attendance to monthly education sessions was associated with greater weight loss (*p* = 0.002) among WbF group (Paskett et al., 2018). More information regarding study design, recruitment procedures, participants’ characteristics, and main outcomes can be found elsewhere (Baltic et al., 2015; Paskett et al., 2018). For the current analysis, we included participants from the intervention arm (WbF) who completed baseline and 12-month measurements on weight, physical activity, dietary intake, and social support (Fig. 1, *n* = 243 out of 426).

### Measurement

Intervention adherence was defined as attendance at the monthly in-person educational sessions, which was recorded by research staff at each church. The percentage of sessions attended was used in the analysis.

Social support from family, friends, and the church were measured at baseline and at 12-months using the Social Support for Eating Habits (SSEH) and the adapted Social Support Inventory for Physical Activity (SSPA) questionnaires (Supplementary Materials) (Dunkel-Schetter, Feinstein, and Call, 1986; Sallis et al., 1987). The SSEH scale is a 10-item self-report questionnaire assessing the frequency of support received from others for healthy eating habits on a scale from 1 (none) to 5 (very often). Subscales of family encouragement and family discouragement were calculated, with higher scores indicating greater encouragement/discouragement. These four subscales were reliable with Cronbach’s alpha of 0.75–0.94 among different populations (Carbonneau et al., 2018; Rieger et al., 2018; Sallis et al., 1987). The SSPA is a 10-item self-reported questionnaire to assess the support received from family/friends and the church on a scale from 1 (never) to 5 (very often). Subscales of SSPA for family/friends and the church family were calculated, with a higher score indicating greater support. The Cronbach’s alpha for the subscales of SSPA range between 0.78 and 0.91, indicating a strong internal consistency (Reis et al., 2011; Smith et al., 2023).

A web-based questionnaire developed by a privately held health and wellness systems company (Viocare, Inc.) was used to assess food frequency and physical activity at baseline and at 12-months. This web-based approach has shown similar performance as the FFQ in paper format, with high intermethod reliability (Kristal et al., 2014). The food frequency questionnaire was used to measure fruit and vegetable consumption at baseline and 12 months (Viocare, 2009). The total consumption of fruit and vegetables (servings/day) and total dietary intake (kcal/week) were calculated. Physical activity was measured using the International Physical Activity Questionnaire (IPAQ) long-form (Sjöström et al., 2002). Participants reported the average days per week and minutes and/or hours per day spent in each of walking, moderate-intensity, and vigorous-intensity physical activities. Time spent participating in each activity per week was multiplied by its typical energy expenditure using metabolic equivalents (METs). The total physical activity (MET·min/week) and walking (MET·min/week) were calculated.

To collect anthropometric measures, at each church a standardized protocol was used by research staff. Height was measured at baseline, weight and waist and hip circumference were measured at baseline and 12-months. BMI (kg/m^2^) was calculated by body weight (kg) and height (m). Weight loss outcomes included the absolute change in weight, BMI, and waist-to-hip ratio.

Other characteristics, such as age, sex, education (high school or less, some college, college graduate or higher), marital status (married/living with a partner, divorced/separated/ widower, never been married), and employment (fulltime/part-time/student, unemployment, retired) collected from paper and telephone-based surveys were included in the analysis as potential confounders.

### Statistical analysis

Descriptive tables of participant characteristics were reported using means/standard deviations (SD) for continuous variables and frequencies for categorical variables. Summary tables of changes in weight loss outcomes, health behaviors, and social support from baseline to 12-months were reported and adjusted for the randomized region.

Due to the small changes in social support, to determine the mediation effect, the potential mediators, change in social support for eating habits (SSEH) and physical activity (SSPA), were dichotomized to SSEH-increased family encouragement (Y/N), SSEH-decreased family discouragement (Y/N), and increased SSPA (Y/N), respectively. Structural equation modeling was used to estimate direct, indirect, and total effects (Baron and Kenny 1986). Each structural equation model included two paths: the first path assessed the relationship between the mediator (e.g., SSPA) and the independent variable (intervention adherence) and controlled for covariates; the second path assessed the outcome variable (e.g., total physical activity) controlled for both the mediator (e.g., SSPA) and the independent variable (intervention adherence) and adjusted for covariates. Inference with standard errors and confidence intervals was performed using Bootstrap methods to determine whether the effect of the mediator was significant (Bollen and Stine 1990). We calculated the proportion of the effect of attendance at the education sessions on increased total physical activity, walking, improved fruit and vegetable intake, and total caloric intake that was mediated by SSEH/SSPA as the ratio of the indirect effect to the total effect (VanderWeele, 2013). To account for potential confounding, all models were adjusted for age, sex, marital status, education, and employment, and geographic region.

Linear regression models were used to quantify the dose-response relationship between changes in total physical activity, fruit and vegetable intake, calories intake, and changes in absolute weight, waist-to-hip ratio, and BMI. Changes in physical activity (min/week) and caloric intake were modeled as continuous variables using linear, quadratic, and restricted cubic splines. Cubic splines can accommodate nonlinearity and provide statistically efficient and visually intuitive descriptions of associations (Harrell Jr, 2015). The likelihood ratio test was used to compare the goodness-of-fit from quadratic and restricted cubic splines models to the linear model. This study included various statistical tests without adjusting for multiple comparisons. Adjustment for multiple comparisons is designed to avoid potentially spurious findings to maintain strict type I error control. However, in exploratory studies, strict type I error control decrease power for associations that are not null, which could miss possibly important findings worth of future investigation (Rothman, 1990a). The analyses reported here were exploratory, with a focus to examine whether social support (SSEH and SSPA) mediated the association between intervention attendance and behavioral changes (fruit and vegetable intake, physical activity) among Appalachian residents since few studies had previously done so. Statistical tests were conducted with significance set at *p* < 0.05. All analyses were completed between November 2021 and May 2022 using STATA IC 17.1 (StataCorp LLC, College Station, TX).

## Results

Among the 243 participants included in the analysis, average age was 57.9 years, 76.2% were female, most were White and married or living with partner (Table 1). More than half of participants were employed full-time, part-time, or were students, and 30.3% were retired. According to the self-reported survey, 72.4% participants were never smokers, 24.3% were former smokers, and 3.3% were current smokers. Using standard measured weight, 62.8% of participants had obesity and 37.2% were overweight. Compared to participants included in the analysis, participants who were excluded due to missing data were younger (52.2 vs. 57.9 years, *p* < 0.001), less likely to have a college or higher degrees (29.4% vs. 41.8%, *p* = 0.03), retired (19.2% vs. 30.3%, *p* = 0.03), and less likely to have public health insurance (22.0% vs. 33.5%, *p* = 0.02). Participants who were excluded from the analysis were more likely to have obesity (73.4% vs. 62.8%, *p* = 0.02).

**Table 1:** Baseline characteristics of Walk by Faith intervention group, participants included vs. excluded

		Participants Included (*n* = 243)		Participants Excluded (*n* = 183)		*P*
		*n*	%	*n*	%	
Age, Years, Mean ± SD		57.94 ± 12.25		52.16 ± 13.05		< 0.001
Sex						0.31
	Male	57	23.8%	50	28.2%	
	Female	182	76.2%	127	71.8%	
Race						0.84
	White	233	97.5%	172	97.2%	
	African American	6	2.5%	5	2.8%	
Education						0.03
	High school or less	58	24.3%	50	28.2%	
	Some College	81	33.9%	75	42.4%	
	College or higher	100	41.8%	52	29.4%	
Marital Status						0.09
	Married/Living with Partner	194	81.2%	128	72.3%	
	Divorced/Separated/Widowed	37	15.5%	38	21.5%	
	Never Been Married	8	3.4%	11	6.2%	
Employment						0.03
	Fulltime/Parttime/Student	139	58.4%	114	64.4%	
	Unemployment	27	11.3%	29	16.4%	
	Retired	72	30.3%	34	19.2%	
Insurance						0.02
	Uninsured	8	3.4%	11	6.2%	
	Public	80	33.5%	39	22.0%	
	Private	151	63.2%	127	71.8%	
Smoking Status						0.67
	Never	173	72.4%	121	68.4%	
	Former	58	24.3%	49	27.7%	
	Current	8	3.3%	7	4.0%	
BMI Category						0.02
	Overweight: 25–29.9 kg/m^2^	89	37.2%	47	26.6%	
	Obesity: ≥30 kg/m^2^	150	62.8%	130	73.4%	

SD: standard deviation

BMI: body mass index

At baseline, among the included participants, average BMI was 32.8 ± 6.0 kg/m^2^ and the average waist-to-hip ratio was 0.9 ± 0.1 (Table 2). According to the self-reported data, the average total physical activity was 5487.8 ± 4565.7 MET min/week, the average walking activity was 1473.3 ± 1642.4 MET min/week, the average fruit and vegetables intake was 3.0 ± 2.0 serving/day, and the average total calories intake was 2051.8 ± 855.1 kcal/day. After the 12-month WbF intervention, significant changes were observed in weight (-1.1 ± 0.3 kg, *p* = 0.001, Table 2), BMI (-0.4 ± 0.1 kg/m^2^, *p* = 0.001), and waist-to-hip ratio (-0.01 ± 0.003, *p* = 0.04). Although participants did not substantially change physical activity level, increased fruit and vegetables intake (0.4 ± 0.1 serving/day, *p* = 0.001) and reduced total calories intake (-322.9 ± 42.2 kcal/day, *p* < 0.001) were observed. We also observed decreased discouragement from family in SSEH (-0.68 ± 0.29, *p* = 0.02) and increased SSPA from church family (1.2 ± 0.5, *p* = 0.01).

**Table 2:** Baseline and 12-month weight, BMI, waist-to-hip ratio, physical activity, dietary intake, and social support measures

Variables	Baseline	12-month	Δ Baseline to 12-month*		
	Mean ± SE	Mean ± SE	LS Mean ± SE	95% CI	**P**
Weight, kg	90.2 ± 18.6	89.3 ± 19.1	-1.13 ± 0.33	-1.77, -0.49	0.001
BMI, kg/m^2^	32.8 ± 5.98	32.5 ± 6.31	-0.40 ± 0.12	-0.63, -0.17	0.001
Waist-to-hip Ratio	0.90 ± 0.09	0.89 ± 0.08	-0.01 ± 0.01	-0.01, -0.001	0.04
Physical Activity, MET·min/Week	5487.8 ± 4565.7	5762.5 ± 5086.0	228.4 ± 279.7	-319.9, 776.7	0.41
Physical Activity, Walk MET·min/Week	1473.3 ± 1642.4	1676.7 ± 1630.3	212.6 ± 112.1	-7.18, 432.4	0.06
Fruit/Vegetables, Servings/Day	2.9 ± 1.98	3.41 ± 2.30	0.44 ± 0.13	0.18, 0.71	0.001
Total Calories, kcal/Day	2051.8 ± 855.1	1734.2 ± 724.0	-322.9 ± 42.2	-405.6, -240.3	< 0.001
SSEH, Family Encourage	11.1 ± 5.24	11.0 ± 5.39	-0.08 ± 0.34	-0.75, 0.59	0.81
SSEH, Family Discourage	10.8 ± 4.84	10.1 ± 4.28	-0.68 ± 0.29	-1.25, -0.12	0.02
SSPA, Family/Friends	26.1 ± 7.16	26.7 ± 6.82	0.49 ± 0.55	-0.59, 1.57	0.37
SSPA, Church	19.1 ± 6.0	20.4 ± 6.37	1.23 ± 0.46	0.34, 2.12	0.007

*Adjusted for geographic region (group randomization)

LS Mean: Least Square mean; SE: standard error

BMI: body mass index

SSEH: social support for eating habits

SSPA: social support for physical activity

When examining the association between intervention attendance and change in social support, we found that each 10% increase in intervention attendance was associated with a higher odd of SSEH-increased encouragement from family (OR = 1.16, 95% CI: 1.04–1.29, Table 3) and increased SSPA from church family (OR = 1.30, 95% CI:1.14–1.47).

**Table 3:** Association of each 10% increase in intervention attendance and change in social support

Changes in SSPA and SSEH	Odds Ratios	95% CI	*P*
SSEH, Increased Family Encourage	1.16	1.04, 1.29	0.008
SSEH, Decreased Family Discourage	1.03	0.93, 1.14	0.62
Increased SSPA, Family/Friends	1.03	0.93, 1.15	0.57
Increased SSPA, Church	1.30	1.14, 1.47	< 0.001

*All models adjusted for age, sex, marital status, education, employment, and geographic region

Analysis of the association between intervention attendance and changes in health behaviors mediated through social support (Table 4) revealed the direct effect of intervention adherence on walking was 10.1 ± 4.6 MET min/week when adjusted for increased SSPA of family/friends (*p* = 0.03) and 13.2 ± 5.1 MET min/week when adjusted increased SSPA of church family (*p* = 0.01). When accounting for changes in social support as potential mediators, the indirect effect of intervention attendance on walking through SSPA of family/friends (*p* = 0.39) and church family (*p* = 0.97) were not significant. The total effect of intervention adherence on walking was 10.6 ± 4.5 MET min/week (*p* = 0.02) and 13.1 ± 4.7 MET min/week (*p* = 0.005) when adjusted for increased SSPA of family/friends and increased SSPA of church family, respectively.

**Table 4:** The association between intervention attendance and changes in health behaviors mediated through social support

Attendance →Δ Behaviors Potential Mediator		Direct Effect			Indirect Effect			Total Effect		
		LS Mean ± SE	95% CI	**P**	LS Mean ± SE	95% CI	**P**	LS Mean ± SE	95% CI	**P**
Intervention Attendance Δ Total Physical Activity MET·min/Week										
	SSPA, Family/Friends	7.62 ± 11.79	-15.49, 30.74	0.52	1.12 ± 1.21	-1.26, 3.49	0.36	8.74 ± 11.70	-14.19, 31.68	0.46
	SSPA, Church	11.03 ± 11.62	-11.75, 33.82	0.34	-2.30 ± 2.77	-7.73, 3.14	0.41	8.74 ± 11.35	-13.52, 30.99	0.44
Intervention Attendance Δ Walking MET·min/Week										
	SSPA, Family/Friends	10.14 ± 4.56	1.20, 19.07	0.02	0.48 ± 0.56	-0.61, 1.57	0.39	10.62 ± 4.48	1.83, 19.40	0.02
	SSPA, Church	13.18 ± 5.06	3.25, 23.11	0.009	-0.04 ± 1.11	-2.21, 2.13	0.97	13.14 ± 4.73	3.87, 2.41	0.005
Intervention Attendance Δ Fruit/Vegetables Intake Servings/Day										
	SSEH, Family Encourage*	0.0018 ± 0.0059	-0.010, 0.013	0.77	**0.0029 ± 0.0015**	**0.00003**,** 0.0058**	**0.05**	0.0047 ± 0.0058	-0.0067, 0.0160	0.42
	SSEH, Family Discourage	0.0022 ± 0.0051	-0.008, 0.012	0.67	-0.00001 ± 0.0001	-0.00023, 0.00021	0.94	0.0022 ± 0.005	-0.0078, 0.012	0.67
Intervention Attendance Δ Calories Intake kcal/Day										
	SSEH, Family Encourage	-0.65 ± 1.97	-4.51, 3.22	0.74	-0.21 ± 0.31	-0.82, 0.41	0.51	-0.85 ± 1.93	-4.63, 2.92	0.66
	SSEH, Family Discourage	-0.90 ± 1.80	-4.43, 2.63	0.62	0.04 ± 0.15	-0.25, 0.34	0.77	-0.85 ± 1.82	-4.42, 2.71	0.64

* % total effect mediated = 61.7%

Using SEM, use dummy variables for categorical variables

**Fig. 2:**
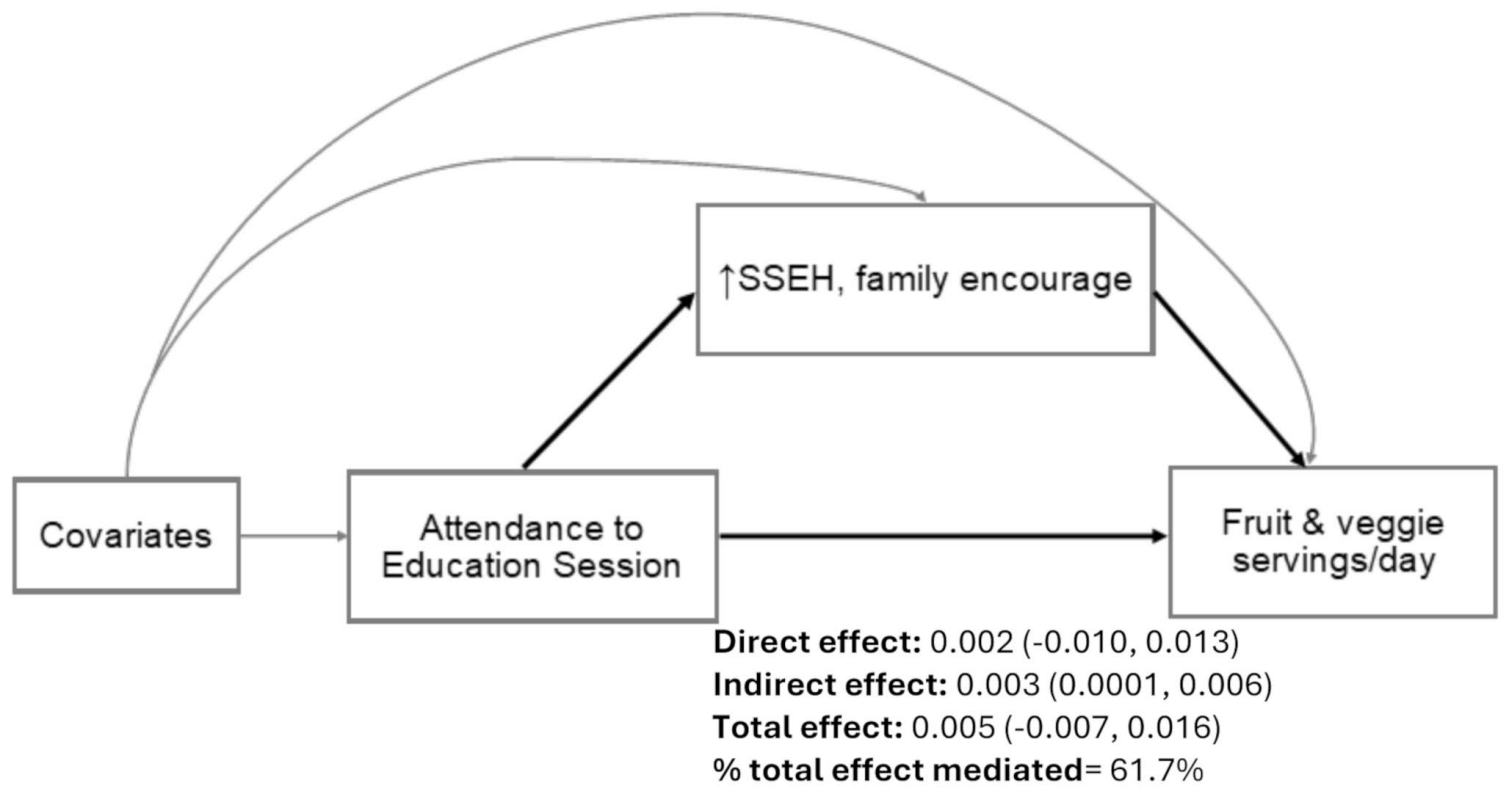
Mediation effect of social support of healthy eating on the association between intervention attendance and fruit & vegetable intake

The direct effect of intervention adherence on fruit and vegetable intake was 0.002 ± 0.006 servings/day adjusted for SSEH-increased family encouragement (*p* = 0.76) and 0.002 ± 0.005 servings/day adjusted for SSEH-decreased family discouragement (*p* = 0.67). When accounting for changes in SSEH as the potential mediators, the indirect effect of intervention attendance on fruit and vegetable intake through SSEH-increased family encouragement was significant (*p* = 0.05, Fig. 2), however, the indirect effect through SSEH-decreased family discouragement was not significant (*p* = 0.94). Increased family encouragement mediated 61.7% of total effect between intervention adherence and fruit and vegetable intake. The direct effect, indirect effect, and total effect of intervention adherence on total physical activity and total calorie intake when adjusted for changes in SSPA and SSEH were not significant.

**Fig. 3:**
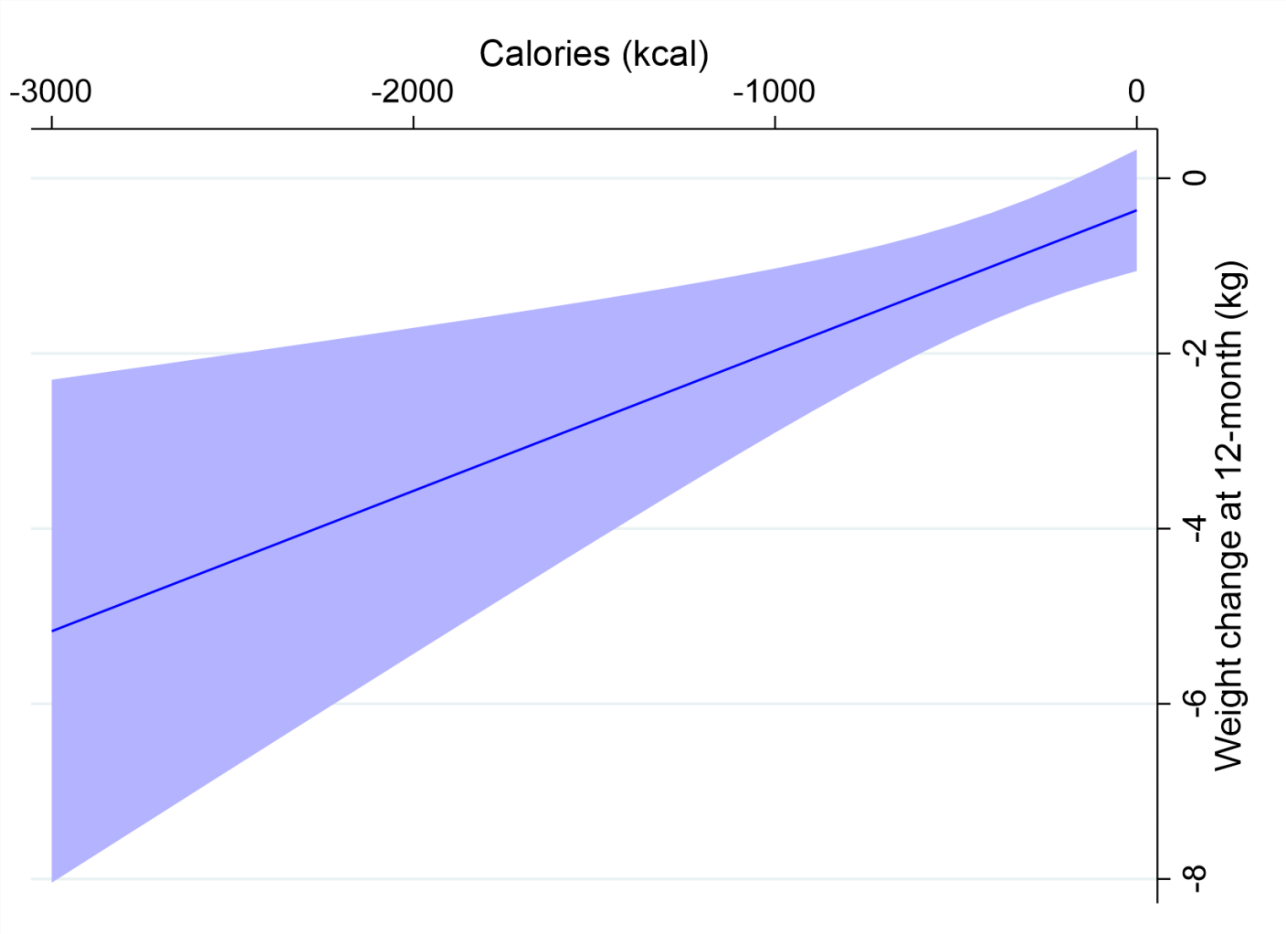
Dose-response relationship between changes total calorie intake (kcal/day) and weight loss at 12-month With every 100 kcal/day decrease, body weight decreased 0.16 ± 0.05 kg (*p* = 0.003)

In terms of the effect of changes in total physical activity, walking, or fruit and vegetable intake on weight, BMI, and waist-to-hip ratio, there was no dose-response relationship (Table 5). However, with every 100 kcal/day decrease in calorie intake, we observed a 0.14 ± 0.05 kg weight loss (*p* = 0.004). The likelihood-ratio (LR) test suggested a cubic spline relationship between changes in caloric intake and weight loss may exist (*p* = 0.09, Fig. 3). After controlling for total physical activity, the linear relationship between caloric intake and weight loss remained significant (Least Square Mean: -0.16 ± 0.05, *p* = 0.003). Similarly, there was a dose-response relationship between changes in caloric intake and changes in BMI: with every 100 kcal/day decrease, BMI decreased 0.06 ± 0.02 kg/m^2^ (*p* = 0.002). The LR test suggested that a cubic spline relationship between changes in caloric intake and BMI may exist (*p* = 0.09). After controlling total physical activity, the linear relationship between caloric intake and changes in BMI remained significant (LS Mean: -0.06 ± 0.02 kg/m^2^, *p* = 0.002).

**Table 5:** The dose-response relationship between changes in health behaviors and weight loss outcomes at 12-month

Weight loss outcomes		LS Mean ± SE	95% CI	*P*
	Change in behaviors			
Weight, kg				
	+ 150 MET·min/Week in Total Physical Activity	-0.01 ± 0.01	-0.03, 0.02	0.78
	+ 150 MET·min/Week in Walking	-0.02 ± 0.030	-0.08, 0.04	0.56
	+ 1 Servings/Day in Fruit/Vegetables	-0.07 ± 0.16	-0.38, 0.23	0.65
	-100 kcal/day in calorie intake, unadjusted ^a^	-0.14 ± 0.05	-0.24, -0.05	0.004
	-100 kcal/day in calorie intake, adjusted ^b^	-0.16 ± 0.05	-0.26, -0.06	0.003
Waist-to-hip Ratio				
	+ 150 MET·min/Week in Total Physical Activity	-0.01 ± 0.01	-0.03, 0.02	0.85
	+ 150 MET·min/Week in Walking	-0.01 ± 0.03	-0.07, 0.05	0.70
	+ 1 Servings/Day in Fruit/Vegetables	-0.14 ± 0.14	-0.42, 0.15	0.34
	-100 kcal/day in calorie intake, unadjusted	-0.07 ± 0.05	-0.16, 0.02	0.16
	-100 kcal/day in calorie intake, adjusted ^b^	-0.05 ± 0.05	-0.15, 0.05	0.35
BMI, kg/m^2^				
	+ 150 MET·min/Week in Total Physical Activity	-0.01 ± 0.01	-0.01, 0.01	0.77
	+ 150 MET·min/Week in Walking	-0.01 ± 0.01	-0.03, 0.01	0.46
	+ 1 Servings/Day in Fruit/Vegetables	-0.02 ± 0.06	-0.13, 0.09	0.76
	-100 kcal/day in calorie intake, unadjusted ^c^	-0.06 ± 0.02	-0.09, -0.02	0.002
	-100 kcal/day in calorie intake, adjusted ^b^	-0.06 ± 0.02	-0.10, -0.02	0.002

All model adjusted for age, sex, marital status, education, employment, and geographic region

^a^ Cubic spline model vs. linear model LR test: *p* = 0.085

^b^ Additional adjusted for total physical activity MET·min/Week

^c^ Cubic spline model vs. linear model LR test: *p* = 0.093

## Discussion

This study examined the role of social support in behavioral changes and corresponding weight loss outcomes among participants from a faith-based weight loss intervention in Appalachian areas. We found that improved social support for healthy eating from family mediated the association of intervention attendance with increased fruit and vegetable intake. Moreover, we observed dose-response relationships between decreased caloric intake and reduced weight and BMI at 12-months.

In behavioral changes, social support represents the resource that influences the cognitive recognition of barriers, sociocultural impediments, and facilitation of coping and recovery strategies (Schwarzer and Knoll 2007). Social support is particularly important for Appalachian residents since they valued Appalachian identity with dietary patterns that emphasize meat, biscuits, and fried foods over vegetables (Shortridge, 2005; Borak et al., 2012; Wenrich et al., 2012). As a result, the most common barrier to following a healthy diet is lack of social support (Brown and Wenrich 2012; Wenrich et al., 2010). The Walk by Faith intervention included a focus on improving social support, especially from the church family, with the goal of increasing physical activity levels (Baltic et al., 2015). As a result, we found that participants who attended more education sessions were more likely to report increased social support from the church family for physical activity and increased social support for healthy eating from family. However, we did not observe any association between changes in social support for physical activity and changes in physical activity. This might be attributable to the non-significant changes in walking or total physical activity level over the 12-month in the WbF intervention. It is possible that individuals who were willing to participate in the behavioral intervention had higher social support at baseline that contributed to smaller changes over time. Future studies should consider including group sessions that involve family and friends, such as virtual 5 K run/walks and hiking trips, to improve the intervention effect on social support for physical activity.

Among Appalachian residents, dietary intake is highly influenced by family values, traditions, and norms (Goins, Spencer, and Williams 2011; Schoenberg et al., 2013). Our study demonstrated that increased family encouragement mediated 62% of the association between intervention attendance and increased fruit and vegetable intake. This finding highlights the importance of social support from family in promoting healthy eating, especially in Appalachian regions. Future interventions to improve healthy diet in this population should incorporate strategies to improve social support from family. For example, programs to involve multiple family members, such as family cooking classes, family grocery shopping trips for healthy food, and community gardening, may increase social support from the family, which could improve intervention efficacy for healthy eating. These strategies have been effective in achieving meaningful behavioral changes among Black and Hispanic women engaged in interventions with features to enhance social support (O’Neal et al., 2022; Crookes et al., 2016; Brown et al., 2021).

Although our participants did not lose a substantial amount of weight over the 12-month intervention, we quantified a dose-response relationship between decreased caloric intake and weight loss. Numerous studies have examined the effect of different dietary patterns (e.g., low-fat, low-carbohydrate, high-protein, and any of these combinations) on weight loss with inconclusive findings (Foster et al. 2010; Gardner et al., 2018; Sacks et al., 2009; Johnston et al. 2014). Few studies examined the dose-response relationship between reduced caloric intake and weight loss outcomes. One systematic review and meta-analysis of randomized controlled trials found that a very-low-calorie diet (< 800 kcal/d) and a low-calorie diet (< 1200 kcal/d) resulted in a 12.3 kg weight loss over a median of 8 weeks (Johansson et al., 2014). However, this study did not quantify the incremental magnitude between reduced caloric intake and weight loss. The dose-response relationship between behavioral changes and weight loss outcomes is an essential aspect of behavioral studies as it can help identify the optimal dose for each behavioral component and provide clinically meaningful benefits to weight and health outcomes. Thus, the observed dose-response effect of caloric intake and weight loss outcomes in Appalachian populations offers valuable information to design future behavioral weight loss studies.

There are several strengths of this study. First, the Walk by Faith Trial included Appalachian residents from five states. Appalachian residents experience a higher burden of obesity and cancer and lack of access to recreation facilities and a healthy diet. These populations also have limited social support from family and friends to promote a healthy diet and exercise (Johnson et al., 2014; O’Brien et al., 2014; Hardin-Fanning, 2013). The Walk by Faith Intervention included a social support component to help participants exercise more and eat healthier. Second, this study collected theoretical behavioral factors, social support for eating habits and physical activity. This allowed us to be the first to examine the role of social support on behavioral changes in Appalachian populations. Understanding these relationships can help identify specific aspects (e.g., social support for healthy eating from family) that could be improved and inform future studies to include tailored strategies needed to promote healthy behaviors in this population.

Our study, however, is subject to some limitations. Participants from this study were regular churchgoers and volunteered to participate in a weight loss program. Thus, our findings may not be generalized to a broader Appalachian population. Although we had 426 participants assigned to the Walk by Faith group, we only included 243 (57.0%) of them in the analysis, excluding 183 due to incomplete data. There were some differences between those who were included and excluded from the study (e.g., age, education, employment, insurance, BMI). It is possible that participants who were included in the study (i.e. had complete data) had positive experiences and success in terms of behavioral changes and weight loss, compared to those participants who were excluded from these analyses because of missing data. The data was missing not at random, which reduced the statistical power and could have introduced biased results. Therefore, when interpreting the results, caution is necessary since the findings may not represent the entire study population. Future studies should consider implementing strategies to reduce missing data, potentially by using more accessible data collection techniques and incentives to ensure more robust and reliable study outcomes. Additionally, we conducted eight mediation analyses but only identified one significant mediator. We recognize that the current study included extensive analyses without adjusting for multiple comparisons, which may limit the robustness and replicability of the results. Adjusting for multiple comparisons is intended to avoid potentially spurious findings when the null hypothesis is true (i.e., to maintain strict type I error control). However, due to the exploratory nature of the current study, strict type I error control may decrease power for associations that are not null (i.e., increasing type II error), which could miss possibly important findings worth of future investigation (Rothman, 1990b). The primary focus of the analyses reported here was to explore the mediation effect of social support on behavioral changes in an Appalachian population. Consequently, our findings should be treated as preliminary and further research is needed to replicate the results in larger, more diverse populations. Additionally, only the Walk by Faith group completed social support measurements, which precluded us from examining the intervention effect on these theoretical-driven behavioral factors by comparing these factors in the attention control group. Lastly, the weight loss intervention did not provide strategies for portion control, counting calories, or other techniques to make healthy food choices and overcome barriers. This may explain the small magnitude of change in total caloric intake and weight loss at 12-month.

Despite these limitations, our study demonstrated the mediation effect of social support for healthy eating on the association between intervention attendance and fruit and vegetable intake. We also quantified a dose-response relationship between decreased total caloric intake and weight loss outcomes. Our findings underscored the critical role of social support and caloric intake among Appalachian populations in losing weight. Future studies should incorporate strategies to increase social support, with dietary modification focused on caloric intake, to improve intervention efficacy for weight loss in Appalachian populations.

## Data Availability

Materials used to conduct the study are not publicly available. De-identified data from this study are not available in a public archive. De-identified data from this study will be made available (as allowable according to institutional IRB standards) by emailing the corresponding author.
